# Exploring the Modus Operandi of Coaches Who Perpetrated Sex Offenses in Canada

**DOI:** 10.3389/fpsyg.2022.856798

**Published:** 2022-04-13

**Authors:** Elisabeth St-Pierre, Sylvie Parent, Nadine Deslauriers-Varin

**Affiliations:** ^1^Department of Physical Education, Laval University, Quebec City, QC, Canada; ^2^Research Chair on Security and Integrity in Sport, Laval University, Quebec City, QC, Canada; ^3^Interdisciplinary Research Center on Intimate Relationship Problems and Sexual Abuse, Montreal, QC, Canada; ^4^School of Social Work and Criminology, Laval University, Quebec City, QC, Canada; ^5^International Center for Comparative Criminology (ICCC), Montreal, QC, Canada

**Keywords:** sexual abuse, modus operandi, coaches, sex offenders, athletes, sports

## Abstract

This study investigated the modus operandi strategies employed by 120 coaches who committed sexual abuse toward 331 athletes under their authority. More than 2,000 Canadian court judgements and media reports were identified using online search databases. Using descriptive analysis, 51 strategies used in six modus operandi stages were identified. Results highlighted the most frequent strategies used by coaches for each stage of the crime commission process. Additionally, findings revealed the influence of the victims' gender, coaches' sport level and year of coaches' first offenses on modus operandi strategies used. Implications for crime prevention measures are discussed.

## Introduction

While sport is known to have a beneficial impact on the mental and physical health of millions of children and adolescents in North America (Canadian Fitness Lifestyle Research Institute, 2013; Noble and Vermillion, 2014), unfortunately, young athletes also experience abuse in this context. Studies reported prevalence of sexual abuse (SA) by a coach ranging from 0.3 to 9.7% (Toftegaard Nielsen, 2001; Leahy et al., 2002; Alexander et al., 2011; Parent et al., 2016). Although other stakeholders have been identified as perpetrators of SA, more intrusive behaviors of sexual violence (e.g., sexual abuse with contact) as well as higher rates of long-term post-traumatic and dissociative symptoms are reported by athletes when the coach is the offender, compared to other perpetrators (e.g., peer athletes) (Leahy et al., 2004; Vertommen et al., 2017). It has been suggested that perpetrators in positions of authority are more likely to use emotionally manipulative strategies in their modus operandi (MO), which may lead to more severe and long-lasting SA and therefore, result in more negative psychological impact (Leahy et al., 2004; Vertommen et al., 2017). However, to this day, coaches' MO remains vastly understudied. The few existing studies on the topic have focused exclusively on strategies involved in one specific stage of MO, namely gaining victim cooperation in SA (Toftegaard Nielsen, 2004; Brackenridge et al., 2008). To our knowledge, no studies have yet documented all MO strategies and stages of SA perpetrated by coaches against one or more athletes under their authority.

### Crime Script and MO

The concept of modus operandi refers to a perpetrator's “behaviors prior to, during, and following sexual abuse” (Kaufman et al., 1998, p. 350). It can be divided into several stages (e.g., gaining the victim's trust, maintaining silence; Leclerc et al., 2009a). Cornish (1994) developed the script model in order to gain a thorough understanding of the various MO stages and strategies involved across the whole crime commission process. Drawing from the script model, it is also possible to pinpoint facilitating conditions and suggest interventions points for prevention tailored for each MO stages of a specific crime. Prior studies have adapted the script model to investigate the MO of sex offenders in general (e.g., Beauregard et al., 2007a; Deslauriers-Varin and Beauregard, 2010; Cook et al., 2018; Chiu and Leclerc, 2019) and child sexual offenders specifically (e.g., Leclerc et al., 2011, 2013; Chopin and Beauregard, 2020). These studies also showed that perpetrators may adapt their MO according to situational variables (e.g., the context in which SA occur), victim characteristics, offender-victim interactions as well as preventive measures that are implemented (e.g., Beauregard et al., 2007b; Cusson, 2007; Deslauriers-Varin and Beauregard, 2010; Deslauriers-Varin and Blais, 2019; Chopin and Beauregard, 2020). To date, the script model has never been applied to investigate SA perpetrated in institutional contexts such as sports. Given that sex offenders in institutions have access to many children and adolescents as part of their work and are often in a position of authority, it is possible that their MO differs from sex offenders' MO in other contexts (e.g., intrafamilial, extrafamilial).

### MO of Perpetrators Working in Institutions

Prior literature has mainly examined sex offenders in institutional contexts as a general group composed of individuals working in various institutions (e.g., schools, sport clubs, churches). Some previous studies on this topic focused specifically on grooming, which is defined as a “process by which a perpetrator isolates and prepares an intended victim” (Brackenridge, 2001, p. 35). Manifestations of grooming include only non-violent strategies such as providing privileges and developing a friendship with a victim whereas the MO covers both non-violent and more violent strategies such as the use of force, coercion, and blitz attack (Cense, 1997; Brackenridge, 2001; Brackenridge et al., 2008; Lanning and Dietz, 2014). Considering that the MO incorporates all behaviors, stages and strategies in the whole crime commission process, grooming specific stages and strategies are included under this concept. In general, studies have shown that sex offenders working in institutions frequently use non-violent strategies such as giving special attention, spending a lot of time with the victim, offering gifts/privileges in order to gain the victim's trust (Leclerc et al., 2005, 2015; Erooga et al., 2012; Lanning and Dietz, 2014; Leclerc and Cale, 2015). Sullivan and Beech (2004) studied the MO of forty-one “professional” child sex offenders (clergy, teachers, and social workers) who spent time in a treatment center and found that most of them had met the child outside of their workplace (77.5%) or took the child away for a night (67.5%) in order to isolate him/her. Leclerc et al. (2005) interviewed 23 men who had sexually abused children while working in institutions (sports, schools, scouts, etc.). To gain the victim's cooperation, these men were more likely to use non-violent grooming strategies such as gradually desensitizing the child from non-sexual to increasingly sexual touching. Most of these offenders had not used any strategies to keep the victim silent. It has been suggested that the offenders' authority over their victim and/or the victim's feeling toward the abuser might “naturally” deter the child from disclosing the abuse (Leclerc et al., 2005). While previous research provides significant insight on sex offenders working in various institutions, several studies emphasized the importance of investigating the MO and dynamics involved in SA perpetrated specifically in sport (Brackenridge, 2001; Brackenridge et al., 2008; Fasting et al., 2013). It has been previously suggested that characteristics and manifestations of SA in sport may differ from those in non-sporting contexts (Brackenridge, 2001; Parent and Bannon, 2012). Bearing in mind that perpetrators may adapt their MO accordingly to situational factors and that sport has its own subculture, norms and rules (Brackenridge, 2003; Parent and Bannon, 2012), it appears crucial to investigate SA perpetrated in this particular context.

### Coaches' MO

Most studies on SA in sport have focused on victims' risk factors (Vertommen et al., 2016; Ohlert et al., 2020; Parent and Vaillancourt-Morel, 2020) rather than on perpetrators' characteristics and MO strategies used to commit those crimes (Kirby et al., 2000; Leahy et al., 2002; Fasting et al., 2013; Vertommen et al., 2017). The current (limited) body of knowledge on coaches' MO and behaviors is primarily derived from qualitative studies based on anecdotal accounts and case studies of victimized athletes. These studies have mainly focused on grooming, which does not necessarily represent the whole repertoire of MO strategies used by coach perpetrators of SA (Brackenridge et al., 2008). Many athletes have reported, in interviews, that their coach took advantage of certain vulnerabilities (e.g., strained relationships with their parents, mental health issues) to become closer to them (Cense, 1997; Brackenridge and Fasting, 2005; Owton and Sparkes, 2017). They started providing extra attention to a particular athlete (Cense, 1997; Owton and Sparkes, 2017), and became the athlete's confidant (Bisgaard and Støckel, 2019). Some athletes mentioned that their coach knew everything about them (Brackenridge and Fasting, 2005) and compared him to a father figure or even to God (Cense, 1997; Brackenridge and Fasting, 2005; Owton and Sparkes, 2017). Athletes' narratives often depicted the coach as someone who holds an excellent reputation, thus making him “fearfully respected” by everyone (Brackenridge and Fasting, 2005). In some instances, athletes also noted a shift in conversations, with their coach beginning to make sexual remarks and asking questions about their sexuality and intimate relationships (Brackenridge and Fasting, 2005; Owton and Sparkes, 2017). Many athletes reported receiving massages from their coach that gradually evolved to sexual touching (Cense, 1997; Owton and Sparkes, 2017). While some coaches encouraged their victims to remain silent (Brackenridge and Fasting, 2005; Bisgaard and Støckel, 2019), most athletes have not disclosed the abuse due to shame, fear of being kicked out of their sport, not being believed, and positive feelings toward their coach (Cense, 1997). In some accounts, the sport organization was aware of the coach's behavior, but refused to do anything because they needed him to win (Brackenridge, 1997).

To our knowledge, only two quantitative studies have specifically investigated the variety of MO strategies used by coach perpetrators of SA in sport (Toftegaard Nielsen, 2004; Brackenridge et al., 2008). However, these have focused exclusively on one specific stage of the coaches' MO, namely, gaining the victim's cooperation. Toftegaard Nielsen (2004) studied 160 criminal and legal cases involving 40 Danish coaches who perpetrated sexual crimes (e.g., pornography, sexual abuse) between 1980 and 2002. In 90% of the cases, perpetrators used rewards and privileges to gain the victim's cooperation while in 40% of the cases, perpetrators gave alcohol to the athlete. Brackenridge et al. (2008) studied 159 cases of coaches' SA reported in journal articles. Although strategies used by coaches were cross tabulated by victim gender, no statistically significant associations were found in their study due to a lack of information in media reports. However, general trends were highlighted. Among others, they found that coaches who had abused female victims tended to be more “intimate” because they more often relied on seductive strategies (e.g., forming a relationship with their athlete) compared to coaches with male victims who appeared more “aggressive” and resorted to strategies such as showing pornography and giving alcohol in order to gain victims' cooperation. Brackenridge et al.'s (2008) findings, therefore, suggest that MO strategies used by coaches may vary depending on the gender of the victim. However, more studies are needed to confirm these possible associations.

### Influences and Adaptations of Coaches' MO

Previous literature on sex offenders has shown that the gender of the victims, situational variables, offender-victim interactions, and preventive measures may influence the MO of sex offenders (Leclerc et al., 2009b, 2013; Deslauriers-Varin and Beauregard, 2010; Chopin and Beauregard, 2020). Studies on the perpetration of SA in sport were mostly based on anecdotal accounts of female victims. Thus, little is known about patterns involved in the victimization of males (Cense, 1997; Cense and Brackenridge, 2001; Hartill, 2005; Parent and Bannon, 2012) and the possible discrepancies of MO strategies used with female or male victims in sport remain understudied.

The influence of sport level on coaches' MO also remains uninvestigated. Considering that most athletes who recounted their experiences of SA in previous qualitative studies were at the elite sport-level, there is a lack of information on SA perpetrated in grassroots sports. However, it is possible that relational dynamics at play in the coach-athlete relationship in elite levels have an impact on MO strategies used by coaches. For instance, many elite athletes mention being dependent on their coach to achieve success in their sport and willing to do anything to win and please their coach (Fasting and Sand, 2015; Bjørnseth and Szabo, 2018). Elite coaches have usually built a strong reputation and hold an impressive track record. Therefore, because they are seen as “experts”, their methods to achieve success often go unquestioned by other stakeholders (e.g., parents, sport administrators) which gives them significant power over athletes. They often manage to intrude and control almost every aspect of the athlete's sporting and personal life (e.g., nutrition, sleep, clothing, school, relationships; Stirling and Kerr, 2009; Bjørnseth and Szabo, 2018; Wilinsky and McCabe, 2020). Given the particularities of the elite sport context and the peculiar elite coach-athlete relationship, strategies used by elite coaches to perpetrate SA may be different than those of their non-elite counterparts.

Sex offenders may also adapt their MO according to preventive measures that are implemented (Cusson, 2007; Deslauriers-Varin and Blais, 2019). Prevention policies in sport gained momentum in the early 2000s (Brackenridge, 2001). Although not documented at this time, there may be differences in the MO strategies used by coaches before and after the advent of preventive measures. In addition, new technologies (e.g., social media) have recently emerged, creating new opportunities to access and contact potential victims (Sanderson and Weathers, 2020). Thus, it appears crucial in terms of prevention to examine the similarities and discrepancies in coaches' MO across the years.

### Aim of the Study

Taking into consideration that gaining a deeper understanding of the whole MO process is paramount to developing effective prevention measures, more research needs to be conducted on this topic. To our knowledge, no studies have yet examined the whole repertoire of strategies used by coaches to perpetrate SA during all MO stages. Factors that may influence coaches' MO such as victims' gender, sport level and year of first abuse, also remain vastly unexplored. The current study aims to fill these gaps by exploring the MO of coaches who perpetrated SA toward athletes under their authority in a sport context. This study had three specific objectives: (1) to describe the strategies used at each stage of coaches MO; (2) to describe the influence of the victims' gender, coaches' sport level and historical context on the strategies used; and (3) to propose future avenues for prevention.

## Methods

### Procedure

The current study is based on the analysis of court judgments and media reports pertaining to 120 cases of SA perpetrated by coaches in Canada. These documents have been identified as valid sources to obtain information and study sex crimes committed in a sport context (Brackenridge et al., 2008; Vertommen et al., 2017). These include an important amount of verified information and offer the perspective of both victims and perpetrators unlike other sources (e.g., athlete narratives; Fasting et al., 2013). Moreover, court rulings are based on legal terminologies of SA, which provide clear definitions of what constitutes abuse and offers the opportunity to cross-cases in order to investigate the variety of strategies employed by multiple coaches who perpetrated SA under the same legal jurisdiction (Fasting et al., 2013). Hence, to gather relevant cases for this study, an online search was conducted on four publicly available legal databases *(Canadian Legal Information Institute, Lexis Advance Quicklaw, Société québécoise d'information juridique and La Référence)*. The following terms were used, both in English and French, to refine the search results: coach + sex(ual) abuse/assault, coach + sex(ual) abuse/assault + athlete(s), coach + sex(ual) abuse/assault + sport(s), professor/teacher + sex(ual) abuse/assault + athlete(s). To ensure that no judgments issued by Canadian courts in civil or criminal matters were omitted, a verification was made by narrowing the search by province and territory. Subsequently, to pinpoint potentially relevant media-covered cases, a search was performed on the *Online Public Library Eureka* and *Google* using the same terms as those used in the legal databases. When information on coaches' MO were reported in at least one recognized media source, it was deemed reliable for this case to be added to the sample. A search was also conducted using the identified coaches' full names in order to identify all journalistic reports available on these cases and gain additional information on the cases selected to be part of the sample. In sum, ~2,000 court judgments and media reports pertaining to 120 cases of SA perpetrated by coaches in Canada between 1967 and 2020 were thoroughly examined to collect data related to these coaches' MO. Given the public nature of court judgments and media reports, the current study received an exemption from the Research Ethics Board of the institution where the study was conducted. Details that could possibly lead to the victims' identification were not used in the current study.

### Sample

To be included in this study, cases needed to fulfill specific criteria: the perpetrator had to be (1) a male^1^ coach; (2) who committed SA against at least one athlete who was under his authority at the time of the crime; (3) who committed SA with contact (e.g., sexual touching, penetration); and (4) for which information was available in court judgments and/or media reports to clearly describe the MO strategies. When no information on coaches' MO was mentioned in court or journal documents, the case was excluded from the sample. Cases involving a coach working in a non-organized sport context (e.g., physical education teacher, personal trainers) were excluded as well as cases involving SA committed by a non-coaching staff member in a position of authority (e.g., team doctor, referee).

The 120 coaches included in our sample perpetrated their first SA offense on average at 33.6 years old (range = 17–73; SD = 11.4). Altogether, coaches had perpetrated SA against 331 athlete victims, thus, resulting in approximately 2.8 victims per coach (range = 1–17). These athletes were on average 13.6 years old (range = 6–17; SD = 2.2) when they were abused by their coach for the first time. In just over half of the cases (53.3%; *n* = 64), coaches offended only against female victims while in about 44% (*n* = 53) of the cases they only assaulted male victims. Three coaches (2.5%) offended against both female and male victims. Among the 331 victims, 133 (40.2%) were girls while 198 (59.8%) were boys. Interestingly, fewer coaches perpetrated SA against boys, but when they did, they victimized a larger number of them ($\bar{x}\;$ = 3.7 male victims per offender; $\bar{x}\;$ = 2.1 female victims per offender). Most coaches (62.5%) were involved at the regional level at the time of the offenses, and more than half (53%) coached in a “ball or object”^2^ sport (e.g., hockey, soccer). Further, given that sex offenders may adapt their MO strategies accordingly to preventive measures that are implemented (Cusson, 2007; Deslauriers-Varin and Blais, 2019) and that the first programs to prevent sexual exploitation in sport were developed in the 2000s, the sample was separated into two groups: (1) coaches who had perpetrated their first sex offense between 1967 and 1999 and (2) those who did so between 2000 and 2020. In just under half of the cases (45.8%; *n* = 55), coaches' first offense occurred between 2000 and 2020.

### Instrument

Based on previous work in the field of criminology and sport sciences, a coding sheet was developed to capture sociodemographic information on coaches, victims (athletes), as well as on MO strategies. This coding sheet was developed based on the revised versions of the Modus Operandi Questionnaire (MOQ) proposed over the years. The MOQ was originally developed by Kaufman (1991) to highlight the broad-spectrum of sexual offenders' MO behaviors. It follows a crime script framework and, as a result, covers each stage of the crime commission process. Over the years, modified versions of the MOQ were developed to identify behaviors specific to child sexual offenders (Leclerc et al., 2005, 2011; Erooga et al., 2019). Even though, the MOQ has helped researchers gain important insight into the MO of child sexual offenders, it has never been used to investigate coaches who perpetrated sex crimes against young athletes. The coding sheet developed for the current study was therefore adapted to better reflect and integrate the particularities and specificities of SA committed in a sport context. In that regard, Brackenridge (2001) has developed a four-stage grooming process model in which she proposed to include a sport-specific stage to study sex offenders' MO in a sport context. This stage aims to assess MO strategies used by coaches, to develop loyalty and exert control over their athletes. Qualitative studies based on athletes' accounts of abuse at the hands of their coach, were also examined to identify specific MO strategies, thus contributing to the improvement of the study coding sheet (Brackenridge, 1997; Owton and Sparkes, 2017; Bisgaard and Støckel, 2019; Prewitt-White, 2019). Additionally, following a preliminary reading of court judgments and media reports, themes related to coaches' MO that emerged, but were not covered by past research or instrument were also added to the coding sheet. This resulted in a coding sheet including eight sample characteristics variables (see Table 1) and 51 MO strategies divided under six MO categories^3^ (see Table 2): targeting a potential victim, gaining trust, developing dependency and exerting control, isolating the athlete, gaining cooperation, maintaining silence. The 51 MO strategies investigated in the current study will be further described in the following Results section.

**Table 1:** Sample characteristics.

**Variables**	**Mean (SD) range**
Coaches' age at first offense (years)	33.6 (11.4) (17–73)
Athletes' age at first abuse (years)	13.6 (2.2) (6–17)
Number of victimized athletes per coach offender	2.8 (2.8) (1–17)
Period during which coaches perpetrated SAs against athletes under their supervision (years)	5.8 (8.8) (0–48)
Time gap between first SA and first police report (years)	14.1 (14.3) (0–49)
	**Frequency (%)**
Victims' gender	
Female	64 (53.3%)
Male	53 (44.2%)
Female and male	3 (2.5%)
Sport level in which coaches were involved	
Regional	75 (62.5%)
Provincial	20 (16.7%)
National	13 (10.8%)
International	12 (10.0%)
Year at first offense	
1967–1999	65 (54.2%)
2000–2020	55 (45.8%)

**Table 2:** Coaches' modus operandi stages and strategies used.

**Modus operandi stages and strategies used**	**Frequency % (*n*) present/yes**
**Targeting a potential victim (*****n*** **=** **120)**
Targeting a vulnerable athlete (e.g., mental health issues, strained relationships with parents)	44.2% (53)
**Gaining trust (*****n*** **=** **120)**
Befriending the athlete, spending a lot of time with the athlete	69.2% (83)
Befriending the athlete's parents, doing a favor for the athlete's parents	38.3% (46)
Promoting his reputation, expertise, past successes	34.2% (41)
Giving gifts or special permission to the athlete unrelated to sport	30.0% (36)
Making the athlete feel special, “like the chosen one”	21.7% (26)
Using his charisma to charm the athlete, parents, and staff	18.3% (22)
Providing additional and individual training for the athlete	16.7% (20)
Requesting the athlete's services to perform a job	14.2% (17)
Providing benefits, opportunities related to the athlete's sport performances	13.3% (16)
Complimenting the athlete's sports performances	12.5% (15)
Not using any strategy to gain trust	12.5% (15)
**Developing dependency and exerting control (*****n*** **=** **120)**
Not using any strategy to develop dependency and exert control	56.7% (68)
Having an authoritarian coaching style, telling the athlete that he/she needs them to succeed	29.2% (35)
Getting parents to relinquish some or all parental control to the coach	17.5% (21)
Humiliating the athlete individually or in front of other athletes	15.0% (18)
Controlling various aspects of the athlete's personal life (e.g., sleep)	14.2% (17)
Controlling various aspects related to sports practice	13.3% (16)
Discouraging/forbidding romantic relationships and/or spending time with friends, family	12.5% (15)
Living in the same house as the athlete	11.7% (14)
Emotional manipulation, pitting athletes against each other	11.7% (14)
Physically assaulting the athlete (e.g., punching, slapping)	10.8% (13)
**Isolating the athlete (*****n*** **=** **116)**
Taking the athlete to an isolated location other than on the training site or their home	36.2% (42)
Taking or inviting the athlete to their home for sport or non-sport purposes	35.3% (41)
Spending a night with the athlete (e.g., share a hotel room, bed, etc.)	30.2% (35)
Taking the athlete to an isolated location on the training site	27.6% (32)
Perpetrating the abuse when there is at least one witness	21.6% (25)
Perpetrating the abuse when others) are present, but had their view blocked from seeing the abuse	15.5% (18)
Offering to drive the athlete somewhere	14.7% (17)
At least one other person is participating in the abuse (e.g., peer athletes)	12.1% (14)
**Gaining cooperation (*****n*** **=** **120)**
Gradually touching the athlete in an increasingly sexual way	60.0% (72)
Normalizing intimate relationships between coaches and athletes, asking questions of sexual nature	42.5% (51)
Exchanging sexual content with the athlete (e.g., letters, texts, photos)	25.0% (30)
Declaring love, being in a romantic relationship or acting as a “secret” couple	24.2% (29)
Providing alcohol or drugs to disinhibit the athlete	19.2% (23)
Taking advantage of the athlete's sleeping state	16.7% (20)
Initiating sexual contact in the form of play	15.0% (18)
Offering or promising benefits in exchange of sexual favors	15.0% (18)
Other strategies (lying, making up a scenario or excuse, insisting at length)	14.2% (17)
Reassuring the victim that it will be okay, that they are not doing anything wrong	13.3% (16)
Integrating sexual contact into sport practice, pretending abuse is part of training	12.5% (15)
Threatening the athlete with a consequence (e.g., stop coaching)	12.5% (15)
Physically restraining, using force	11.7% (14)
Offering to provide sex education to the athlete	11.7% (14)
Showing sexually explicit material to the athlete	10.0% (12)
**Maintaining silence (*****n*** **=** **120)**
Not using any strategy to silence the athlete	62.5% (75)
Sports organization is aware of the abuse but does not report	25.0% (30)
Asking the athlete not to tell anyone, saying it should remain a secret	17.5% (21)
Other strategies (e.g., asking the athlete to lie, offering gifts or benefits)	17.5% (21)
Threatening the athlete with punishment or making threats about physical safety	16.7% (20)
**Strategies involved in multiple MO stages (*****n*** **=** **120)**
Strategies to avoid detection	24.2% (29)

### Statistical Analysis

In-depth readings of the court judgments and media articles for the 120 selected cases allowed the lead author to code^4^ and analyze sociodemographic and MO strategies variables using IBM SPSS 27.0. The MO strategies were all coded as dichotomous variables (0 = Absent; 1 = Present). In cases involving multiple victims and events of abuse, strategies were coded as Present when the coach had used them at least once (against any victim and in one or more instances). This provided an exhaustive description of all MO strategies used by coaches during their crime commission. First, descriptive analyses were conducted with all sociodemographic and MO variables included in the study. Second, Chi-square analyses were then carried out to investigate if the MO strategies used by coaches varied based on: (1) the gender of victims; (2) sport level, and; (3) year of the coaches' first SA against an athlete. Considering the high number of Chi-square tests performed and to minimize type 1 error, the Bonferroni correction was used.^5^ Given the number of MO strategies included in the present study, only statistically significant group differences, based on the Bonferroni-corrected *p*-value, are presented in the Results section.

## Results

### MO Strategies

The results of this study highlight the prevalence of 51 MO strategies used by coaches during each MO stage (see Table 2). In almost half of the cases (44.2%; *n* = 53) coaches admitted to *Targeting a potential victim* by deliberately selecting a vulnerable athlete as a victim (e.g., mental health issues, prior history of SA, disability). The most common strategies used by coaches during the *Gaining trust* stage were to establish emotional closeness by becoming the athlete's friend or confidant (69.2%; *n* = 83) and to groom parents by befriending them, offering services or financial help (38.3%; *n* = 46). In more than half of the cases (56.7%; *n* = 68), coaches did not use any strategy to *Develop dependency and exert control* over athletes under their supervision. However, about a third of them (29.2%; *n* = 35) had an authoritarian coaching style that made athletes feel intimidated. Strategies involving emotional manipulation such as pitting team athletes against each other (11.7%, *n* = 14) and physically assaulting the athlete (10.8%; *n* = 13) were less often used by coaches. Furthermore, taking the athlete to an isolated location aside from the training site or their home (36.2%; *n* = 42) and taking the athlete to their home for athletic or non-athletic reasons (e.g., showing a book on gymnastic techniques, inviting the athlete to help replace light bulbs) (35.3%; *n* = 41) were the most frequently used strategies during the *Isolation* stage. Surprisingly, some coaches did not offend when they were alone with the victim. In one out of five cases (21.6%; *n* = 25), someone had witnessed the abuse while in 12.1% (*n* = 14) another individual was directly involved in the abuse as a perpetrator or victim. With respect to *Gaining the athlete's cooperation* during the abuse, more than half of coaches gradually touched the athletes in a more sexual way (60.0%; *n* = 72). Quite frequently (42.5%; *n* = 51), coaches had normalized sexuality by making sexual innuendos or asking questions about the athlete's sexuality. Strategies such as offering alcohol or drugs to the victims (19.2%; *n* = 23), bribing the athlete with benefits, privileges, and gifts in exchange for sexual favors (12.5%; *n* = 15) and perpetrating the abuse while manually assisting an athlete in a sport movement (12.5%; *n* = 15) were used less frequently by coaches in our sample to facilitate the abuse. Most coaches (62.5%; *n* = 75) did not use any strategy to *Maintain the silence* of the athlete after the abuse. Interestingly, in 25% of the cases (*n* = 30), sport organizations had been formally or informally made aware of the abuse but did not make a report to authorities. Some coaches (24.2%; *n* = 29) also took special precautions to avoid detection of SA (e.g., locking the door, avoiding cameras, installing deleting messages apps). In some instances, it was used to gain trust while in others it was employed to isolate the victim or to maintain silence.

### MO Strategies and Gender of the Victims

Table 3 shows that the gender of victims had a significant influence on some of the MO strategies used by coaches to commit SA. Three strategies were more likely to be employed with female than male victims: (1) making the athlete feel special, making them feel like the “chosen one” to gain trust [χ^2^(1) = 15.38; *p* < 0.000, Phi = 0.36], (2) exchanging sexual content with their athlete [χ^2^(1) = 16.64 *p* < 0.000, Phi = 0.38], and (3) making a love declaration or being in a romantic relationship with them [χ^2^(1) = 22.95 *p* < 0.000, Phi = 0.44] to gain cooperation. In comparison, two strategies to gain the athletes' cooperation were more likely to be used with male than female victims: (1) taking advantage of the athlete's sleep [χ^2^(1) = 11.72, *p* < 0.000, Phi = 0.32], and (2) initiating sexual contact in the form of play [χ^2^(1) = 12.42, *p* < 0.000, Phi = 0.33]. For all other strategies, no statistical relationship was found suggesting that these strategies were used independently of the victim's gender.

**Table 3:** Comparison of MO strategies used for male and female victims.

**Factors**	**Female victims (*n* = 64)**	**Male victims (*n* = 53)**	**Group comparisons (*n* = 117)**
^**+**^ **MO strategies**			
Making the athlete feel special, “like the chosen one”	35.9%	5.7%	χ^2^(1) = 15.38^*^
			Phi = 0.36
Exchanging sexual content with the athlete	40.6%	7.5%	χ^2^(1) = 16.64^*^
			Phi = 0.38
Declaring love, being in a romantic relationship or acting as a “secret” couple	42.2%	3.8%	χ^2^(1) = 22.95^*^
			Phi = 0.44
Taking advantage of the athlete's sleeping state	6.3%	30.2%	χ^2^(1) = 11.72^*^
			Phi = 0.32
Initiating sexual contact in the form of play	4.7%	28.3%	χ^2^(1) = 12.42^*^
			Phi = 0.33

+ *Bonferroni corrected p-value: 0.05/51 = 0.001*,

* *p < 0.001*.

### MO Strategies and Coaches' Sport Level

Table 4 shows strategies used according to the sport level in which the coach was involved during the period of the abuse. Strategies such as making the athlete feel special [χ^2^(1) = 12.90, *p* < 0.000, Phi = 0.33], complimenting the athletes' sport performances [χ^2^(1) = 10.98, *p* < 0.000, Phi = 0.30], and promoting their expertise [χ^2^(1) = 40.69, *p* < 0.000, Phi = 0.58] to gain trust were significantly more likely to be used by coaches at elite than non-elite levels. Furthermore, encouraging parents to relinquish some or all parental control to the coach [χ^2^(1) = 32.42, *p* < 0.000, Phi = 0.52] and controlling the athlete's personal life [χ^2^(1) = 29.73, *p* < 0.000, Phi = 0.50] to develop loyalty and exert control were strategies more likely to be associated with coaches at elite levels while not using any strategy to exert control was the only strategy employed more often by non-elite coaches [χ^2^(1) = 45.96, *p* < 0.000, Phi = 0.42]. No statistical associations were observed for any of the other strategies, implying that they were used regardless of the sport level.

**Table 4:** Comparison of MO strategies used based on coaches' sport level.

**Factors**	**Regional/provincial**	**National/international**	**Group comparisons**
	**(non-elite) (*n* = 95)**	**(elite) (*n* = 25)**	**(*n* =120)**
^**+**^ **MO strategies**
Making the athlete feel special, “like the chosen one”	14.7%	48.0%	χ^2^(1) = 12.90^*^
			Phi = 0.33
Complimenting the athlete's sports performance	7.4%	32.0%	χ^2^(1) = 10.98^*^
			Phi = 0.30
Promoting his reputation, expertise, past successes	20.0%	88.0%	χ^2^(1) = 40.69^*^
			Phi = 0.58
Getting parents to relinquish some or all parental control to the coach	7.4%	56.0%	χ^2^(1) = 32.42^*^
			Phi = 0.52
Controlling various aspects of the athlete's personal life	5.3%	48.0%	χ^2^(1) = 29.73^*^
			Phi = 0.50
Controlling various aspects related to sports practice	2.1%	56.0%	χ^2^(1) = 49.75^*^
			Phi = 0.64
Emotional manipulation, pitting athletes against each other	4.2%	40.0%	χ^2^(1) = 24.6^*^
			Phi = 0.45
Living in the same house as the athlete	6.3%	32.0%	χ^2^(1) = 12.67^*^
			Phi = 0.33
Having an authoritarian coaching style, telling the athlete that he needs him to succeed	14.7%	84.0%	χ^2^(1) = 45.96^*^
			Phi = 0.62
Not using any strategy develop dependency and exert control	67.4%	16.0%	χ^2^(1) = 21.27^*^
			Phi = 0.42

+ *Bonferroni corrected p-value: 0.05/51 = 0.001*,

* *p < 0.001*.

### MO Strategies and Year of First Abuse

Table 5 highlights the distinction among strategies used by coaches based on the year of their first known offense perpetrated against an athlete under their supervision. Waiting to spend a night with the athlete to isolate the victim [χ^2^(1) = 14.20, *p* < 0.000, Phi = 0.35] was more likely to be employed by coaches who offended for the first time between 1967 and 1999 while exchanging sexual content with their athlete [χ^2^(1) = 31.43, *p* < 0.000, Phi = 0.51] to gain cooperation was more frequently used by those who did so between 2000 and 2020. With regards to all other strategies, no statistical relationship was found, suggesting that the use of these strategies was not influenced by the year of first abuse.

**Table 5:** Comparison of MO strategies used based on year of first offending.

**Factors**	**1967–1999 (*n* = 65)**	**2000–2020 (*n* = 55)**	**Group comparisons (*n* = 120)**
^**+**^ **MO strategies**
Spending a night with the athlete (e.g., share a hotel room, bed, etc.)	45.2%	13.0%	χ^2^(1) = 14.20^*^
			Phi = 0.35
Exchanging sexual content with the athlete (e.g., letters, texts, photos)	4.6%	49.1%	χ^2^(1) = 31.43^*^
			Phi = 0.51

+ *Bonferroni corrected p-value: 0.05/51 = 0.001*

* *p < 0.001*.

## Discussion

### Coaches' MO

This study highlights the MO strategies used by coach perpetrators throughout six MO stages. Our results will be discussed in light of the existing literature and specifically tailored interventions to increase the perceived risk of committing SA will be suggested. As previously observed in studies on child sexual abuse in institutional settings (Leclerc et al., 2005) and in sport (Brackenridge and Fasting, 2005; Owton and Sparkes, 2017; Bisgaard and Støckel, 2019), coaches mainly relied on grooming strategies to gain the athlete's trust and to gain their cooperation during the abuse. Most of them established an emotional proximity with athletes by befriending and spending a lot of time with them (e.g., calling the athlete at all hours of the day, discussing personal matters, taking the athlete out to dinner) to gain their trust. This is congruent with testimonies of victimized athletes that included athletes identifying their coach as their best friend or someone who knew everything about them (Cense and Brackenridge, 2001; Owton and Sparkes, 2017). Given that it is considered normal for coaches and athletes to be in close proximity, whether during informal (e.g., team parties) or formal outings (e.g., competitions), it appeared relatively easy for coaches to gain trust by befriending the athlete without raising any suspicions (Brackenridge et al., 2008).

Once this “trusting” foundation has been laid, it was relatively easy for coaches to take the athlete to an isolated location aside from the training site or coaches' home (e.g., wooden area, isolated parking spot, hotel room). Previous studies in sport have focused on contextual risk factors (e.g., location, context surrounding the abuse) rather than on strategies used to isolate the athlete before the abuse (Kirby and Greaves, 1997; Cense and Brackenridge, 2001; Toftegaard Nielsen, 2004; Brackenridge et al., 2008). Competitions away and social occasions not related to sport were identified in earlier research as contextual risk factors for SA (Kirby and Greaves, 1997; Brackenridge et al., 2008) and coaches in our sample were likely to take advantage of these opportunities to bring the athlete to an isolated area. Future studies should investigate the locations and context associated with the MO of coaches. Further, safeguarding sport policies should clarify the boundaries of the coach-athlete relationship by discouraging coaches to spend time alone with one athlete during activities outside of the sport context. Once alone with the athlete, coaches in our study felt more at ease to shift the nature of conversations toward sexual matters and to gradually touch the athletes in a more sexual way to gain cooperation in the abuse. As previously indicated by Brackenridge (2001) and Kirby and Greaves (1997), some coaches in our sample took advantage of sport-related opportunities (e.g., therapeutical massages, treating an injury) to touch the athlete in an increasingly sexual way. However, these results are in contradiction with Toftegaard Nielsen's (2004) findings that showed a high tendency among Danish coaches to rely on giving rewards and privileges to gain the athlete's cooperation in the abuse. It is also worth noting that some coaches in our sample used more aggressive strategies or purposedly relied on the athlete's altered state of consciousness instead of resorting to grooming strategies to gain the athlete's cooperation in the abuse. For instance, some of them took advantage of the athlete's sleep or provided alcohol, drugs, or medicine—in some cases without the athlete's knowledge (e.g., drink spiking)—to lower the athlete's inhibitions, while others employed more violent strategies such as force or physical restraint to perpetrate the abuse. In that sense, coaches' MO tended to be different than perpetrators in other positions of authority who mainly use grooming strategies. These results also highlight the significance for future studies to focus on all MO strategies used by coaches and not only non-violent “grooming” strategies.

Our results showed that most coaches in our study did not use any strategy to maintain the victim's silence following the abuse. Many athletes in our sample were under the illusion of consenting to the relationship with their coach, which may explain why coaches did not directly ask the victim to remain silent. This is congruent with Leclerc et al.'s (2005) results, who found that perpetrators in institutional contexts often did not feel the need to prevent victims' disclosure due to their authority over their victim and the victim's feelings toward the abuser. As observed in prior studies (Cense and Brackenridge, 2001; Brackenridge and Fasting, 2005), some athletes in our study mentioned the reasons why they kept silent: they felt ashamed, did not believe they were being abused, or feared losing their sport or their coach's attention.

Unfortunately, our results showed that athletes were not the only ones who kept quiet about the abuse. Surprisingly, some sports organizations were aware of the abuse but did not report it. This dynamic may be explained by the fear of false allegations, destroying the coach's reputation, a lack of knowledge regarding mandatory reporting of child sexual abuse, ambiguity over what was deemed abusive behaviors or a fear of backlash (Brackenridge, 2001; Brackenridge and Fasting, 2005; Howard and England-Kennedy, 2006; Parent, 2011). Organizational tolerance such as bystander inaction and a culture of silence were also identified as dominant social factors that are conducive of SA in sport (Roberts et al., 2021). This underlines the importance of investigating strategies used by coaches not only with victims but also with other stakeholders (e.g., parents, colleagues, administrators, medical teams). As pointed out earlier by Bisgaard and Støckel (2019), the grooming process in sports focuses solely on the coach-athlete relationship, therefore omitting to examine the MO strategies employed to manipulate or coerce individuals close to the athlete.

In stark contrast to other perpetrators in positions of authority in institutional settings (i.e., teachers, priests), coaches in our study appeared to use a larger repertoire of strategies to exert control (Sullivan and Beech, 2004; Leclerc et al., 2005; Erooga et al., 2012). Despite the fact that this MO stage has rarely been identified in studies using the script model to capture the whole crime commission process of sex offenders, almost half of our sample used at least one strategy to exert control. Examples include having an authoritarian coaching style that made athletes feel intimidated, encouraging parents to relinquish parental control, humiliating their athletes, controlling their sporting and personal life (e.g., sleep, weight), living in the same house as the athlete, playing mental games by putting athletes in competition with one another and physically assaulting the athlete. Considering that most coaches in our sample were working at the regional or provincial levels rather than at the national or international levels, these strategies may be underestimated in the current study since they have been associated with elite levels of competition. While questionable practices and abuse of power of elite coaches may be tolerated and justified as a way to develop an athlete's full potential (Brackenridge, 2001), it may raise red flags for coaches at non-elite levels to start exerting control when no sport performances are at stake. Given the lack of studies that have been conducted on athletes performing at non-elite levels, this calls upon the need to further investigate the experience of non-elite athletes.

Another interesting finding of our study was that some coaches did not perpetrate the abuse while being alone with the athlete. In some cases, someone witnessed the abuse (21.6%) while in others (11.7%) at least one other person participated in the abuse, the majority of whom were peer athletes. These acts frequently happened while playing a game (e.g., strip poker, fellatio contest) and young athletes were often called upon to perform sexual acts on fellow teammates. The overt nature of these SA often led athletes to see themselves as willing participants. To our knowledge, no studies in sport have yet discussed these strategies. Further, this has rarely been discussed in studies on child sexual offenders in institutional settings. Sullivan and Beech (2004) study showed that some professional perpetrators admitted, during interviews, that on some occasions they perpetrated SA on more than one victim at the same time. These results questioned the presumption that perpetrators always strive to avoid being seen by others when perpetrating SA. In terms of prevention, these findings are interesting because they suggest that coaches will sometimes abuse an athlete while others—who may act as capable guardians—are present. A capable guardian represents “any person who has the capacity to interrupt the crime commission either directly or indirectly” (Leclerc et al., 2015, p. 5). Reynald (2010) identified three factors that help guardians intervene and derail criminal opportunities: the willingness to supervise, ability to identify potential offenders and willingness to intervene. This highlights the importance of educating athletes and parents, as well as all sport stakeholders who can act as a capable guardian.

### Covariates of Coaches' MO

Our results indicated some discrepancies between strategies used with female and male victims. In line with Brackenridge et al. (2008) findings, declaring love to gain the athlete's cooperation in the abuse was significantly associated with female victims. However, in contrast with earlier findings on coach perpetrators (Brackenridge et al., 2008), that showed a tendency for male victims to experience more “aggressive” grooming strategies (e.g., being shown pornography), coaches with male victims in our sample took advantage of the victim's sleep and played sexualized games. Ultimately, waiting for male athletes to be asleep may have been a strategic choice for coaches to avoid any physical confrontation with the athlete prior to the abuse. Considering that males in our study are athletes with potentially strong athletic capabilities, some of them might have physically resisted if awakened, thus increasing the risk for coaches of being unable to complete the abuse. With regards to initiating sexualized games, some of these were instigated as a form of diversion and aimed to disguise the abuse (e.g., squabbling with the athlete) while others were blatantly sexual in nature (e.g., playing strip poker). Although not often discussed in prior literature on child sexual offenders, these games served to mask the abuse by making it seem non-threatening and somehow fun for the child (Pryor, 1996; Martschuk et al., 2018). In our study, other individuals (peer athletes, friends, etc.) were sometimes witnessing or directly involved in those games, thus increasing the perceived normality of them. These strategies used with male victims have yet to be discussed in the literature related to SA in sport. Considering the differences between coaches' MO with male and female victims and the lack of information on male victims of SA, future studies should investigate male victims specifically and consider the possible influence of victims' gender on strategies used by coaches to perpetrate SA.

Our results also showed that coaches' sport level influenced strategies aimed to gain trust and exert control. While not using any strategy to exert control was associated with non-elite coaches, strategies directly linked to sport were more likely to be employed by elite coaches. They relied on strategies such as complimenting the athlete's sport performance and promoting their coaching expertise, reputation, and past successes to gain trust. Most of the time, this resulted in athletes and parents worshiping and admiring the coach, with some of them even comparing them to God or a king. Once elite coaches had built trusting relationships, they were able to slowly encourage parents to relinquish some form of parental authority to them and gradually took over many areas of an athlete's life such as their diet/weight, sleep, clothing, education, social life, or love/sex life. This results in the isolation of an athlete from any support system outside of sport, thus reinforcing the power of the coach and making it easier to control and abuse them (Brackenridge and Kirby, 1997; Cense and Brackenridge, 2001; Wilinsky and McCabe, 2020). In more extreme cases, some elite coaches lived in the same house as their athletes, thus giving them full unrestricted access to their victims. This highlights the extent of power that elite coaches hold over their athletes, which perhaps may facilitate the perpetration of SA. In that sense, MO strategies based on control used by elite coaches differ from those of non-elite coaches. This is also in contrast with the MO strategies normally observed among offenders in positions of authority in other institutional settings. Perpetrators in a position of power will more often use grooming strategies instead of controlling, violent and authoritative strategies in order to appear non-threatening to the child (Sullivan and Beech, 2004; Leclerc et al., 2005; Lanning and Dietz, 2014). As a result, they can more easily gain the victims' cooperation in SA by avoiding resistance and maintain the on-going abuse for a longer period of time (Lanning and Dietz, 2014). Non-violent grooming strategies are also a strategic choice for offenders to remain undetected, compared to more coercive and controlling strategies that increased the likelihood of victims' disclosure (Kaufman and Patterson, 2010).

Finally, our results suggest that only two MO strategies used by coaches were influenced by the year of coaches' first offense. While coaches who first offended after 1999 have more frequently exchanged sexual content to gain cooperation, those who did prior to 2000 were more likely to wait for an overnight sleep to isolate the victim before the abuse. Rules regarding overnight stays and sleeping arrangements were among the first preventive strategies discussed in action plans and sport literature during the 1990s and early 2000s (Fried, 1996; McKay, 1997; Canadian Hockey Association, 1998; MacGregor, 1998; Brackenridge, 2003). Given that sex offenders may adapt their MO strategies accordingly to preventive measures (Cusson, 2007; Deslauriers-Varin and Blais, 2019), early preventive efforts may have decreased opportunities for coaches to be able to spend a night with the athlete, potentially explaining why coaches were less likely to resort to this strategy after 1999. With respect to exchanging sexual content with athletes to gain cooperation, a recent study showed that social media helps coaches prepare their victims by slowly crossing the athlete's boundaries and by gradually testing their receptivity to sexual activities (Sanderson and Weathers, 2020). When athletes engaged willingly in these exchanges and did not report them, coaches tended to become more comfortable and sent sexual messages, photos or videos that eventually escalated to in-person sexual encounters (Sanderson and Weathers, 2020). Thus, new virtual technologies emerging between 2000 and 2020s may have facilitated the transaction of sexual content between coach and athlete. Establishing clear rules regarding coaches' behaviors online are imperative. Although, making comments and asking questions on one's sexuality is forbidden in current policies, it should be specified that this extends to virtual conversations (Coaching Association of Canada, 2021). Coaches should prioritize group conversations online and refrain from using apps on which pictures and conversations are automatically deleted. Tracking athletes' locations on social media should also be prohibited.

## Limitations

This study provided an important in-depth understanding of the strategies involved throughout multiple stages of the MO of coaches. However, given that a great deal of sex crimes perpetrated in sport never reached the justice system (Brackenridge, 2001; Fasting et al., 2013), cases included in our study only represent the tip of the iceberg. Results should be interpreted with caution considering that these cases have been documented in newspaper articles and court judgments, thus representing only the MO of coaches who have been detected and arrested. It is possible that those who remain undetected, employed other or more effective strategies to avoid being caught. Further, it is possible that coaches withheld or distorted certain information intentionally to avoid harsher sentences. This may be avoided in the future by conducting research interviews with coaches who perpetrated SA in sport. Some evidence may not have been made available publicly and victims may have retained some grueling details. It is also worth noting that journalistic reports may lack key information and only provide details on specific cases or events of SA (Fasting et al., 2013). This study also solely investigated the MO of male coaches and therefore does not reflect the MO of female coaches who perpetrated SA in sport.

## Conclusion and Future Research

The aim of this study was to explore MO strategies of coaches as perpetrators of SA, a neglected area in research on sexual abuse in sport, until now. No prior studies investigated all MO strategies involved in the whole crime commission process of coaches who perpetrated SA in sport against athletes under their authority. As previously observed in studies on child sexual abuse (Leclerc et al., 2005), most coaches in our sample groomed their victim by gradually touching the athlete in an increasingly sexual way to gain cooperation. However, coaches in our study appeared to use a larger repertoire of strategies to exert control compared to other perpetrators in positions of authority in institutional settings (i.e., teachers, priests). Our study results, therefore, suggest that the MO of coaches may be different from the MO of other offenders in positions of authority in institutional settings, thus highlighting the need, importance, and relevance of studying this specific subgroup of offenders. Given that coaches' MO may be influenced by various individual characteristics and situational factors, it may be interesting to study the MO of female coaches. Future studies should also focus on the influence of other variables on the coaches' MO such as the nature of the abuse, location (e.g., training sites, coach's home) and context (e.g., social activities, training sessions, competitions away). Identifying different behavioral profiles of coach sex offenders would also provide an in-depth understanding of the decisions process involved in the crime commission. Moreover, future studies should aim to compare coaches' profiles to those of other individuals in positions of authority in institutional settings (e.g., priests, teachers) to further examine the heterogeneity of behavioral patterns that exists within institutional sex offenders.

## Data Availability Statement

The raw data supporting the conclusions of this article will be made available by the authors, without undue reservation.

## Ethics Statement

The studies involving human participants were reviewed and approved by Laval University Research Ethics Board. Written informed consent for participation was not required for this study in accordance with the national legislation and the institutional requirements.

## Author Contributions

ES-P was responsible for the conception and study design, project management, and wrote the manuscript. SP and ND-V contributed to the project management and reviewed the manuscript. All authors contributed to the article and approved the submitted version.

## Conflict of Interest

The authors declare that the research was conducted in the absence of any commercial or financial relationships that could be construed as a potential conflict of interest.

## Publisher's Note

All claims expressed in this article are solely those of the authors and do not necessarily represent those of their affiliated organizations, or those of the publisher, the editors and the reviewers. Any product that may be evaluated in this article, or claim that may be made by its manufacturer, is not guaranteed or endorsed by the publisher.

## Footnotes

^1^Female coaches were excluded because they were too few and believed to be using a different MO to commit their SA. A follow-up study will look at these cases specifically.

^2^Other coaches in our sample were involved in various types of sport: technical sports (e.g., sailing; 11.3%); endurance sports (e.g., cycling; 9.6%); aesthetical sports (e.g., gymnastics; 11.3%); and combat sports (e.g., judo; 14.8%).

^3^Strategies to avoid detection were classified under “strategies involved in multiple MO stages” because they could not be linked to one specific category or stage of the MO (i.e., was sometimes employed during various stages, such as gaining trust, isolating the athlete, and maintaining silence).

^4^While the coding sheet was validated by all three authors, variables were all coded by the lead author. Sustained discussions however occurred between the lead authors and the second and third author throughout the coding process to ensure its operationalization, reliability and agreement with the current knowledge and procedures in the field. The lead author also coded all variables at two different time points for intracoder reliability.

^5^The 0.05 threshold p-value was divided by the number of tests being performed for each of these three sociodemographic characteristics. As such, the Bonferroni-corrected p-value for the tested associations between all the MO strategies (51) and (1) gender of the victims, (2) sport level, and; (3) year of the first abuse perpetrated were: *p* < 0.001 (0.05/51).
